# Association of intraprocedural near admission-level blood pressure with functional outcome in stroke patients treated with mechanical thrombectomy

**DOI:** 10.1186/s42466-024-00345-0

**Published:** 2024-10-01

**Authors:** Min Chen, Lukas Daniel Sauer, Mika Herwig, Jessica Jesser, Meinhard Kieser, Arne Potreck, Markus Möhlenbruch, Peter Arthur Ringleb, Silvia Schönenberger

**Affiliations:** 1 Department of Neurology, Heidelberg University Hospital, Heidelberg University, Heidelberg, Germany; 2 Institute of Medical Biometry, Heidelberg University, Im Neuenheimer Feld 130.3, 69120 Heidelberg, Germany; 3 Department of Neuroradiology, Heidelberg University Hospital, Heidelberg University, Heidelberg, Germany

**Keywords:** Endovascular stroke treatment, Blood pressure, Periprocedural management

## Abstract

**Background:**

Optimal blood pressure management during endovascular stroke treatment is not certain. We hypothesized that time or proportion of intraprocedural systolic blood pressure spent in a range around admission blood pressure might be associated with better clinical outcome.

**Methods:**

We conducted a retrospective observational study at a single center at a university hospital, which included patients from August 2018 to September 2020 suffering from acute ischemic stroke with anterior circulation vessel occlusion and treated with endovascular therapy. Time and proportion of procedure time where systolic blood pressure (SBP) was near the baseline SBP on admission (bSBP) were used as exposure variables. The primary outcome was the occurrence of mRS score 0–2 three months after stroke. The primary analysis was performed by fitting a logistic regression model adjusted for baseline NIHSS, pre-stroke mRS, mTICI score, intubation, age and sex.

**Results:**

We included 589 patients in the analysis. Mean (SD) age was 76 (12) years, 315 were women (53%) and mean (SD) NIHSS score at admission was 15 (7.5). Mean (SD) bSBP was 167 (28) mmHg and mean (SD) intraprocedural SBP was 147 (21) mmHg. The proportion of time where intraprocedural SBP was in range of bSBP ± 20% was associated with a slightly higher odds of achieving favorable outcome (adjusted OR, 1.007; 95% CI, 1.0003–1.013).

**Conclusion:**

A higher proportion of intraprocedural time with systolic blood pressure in range of ± 20% of the admission level is associated with higher odds of favorable functional outcome.

**Trial Registration:**

Not applicable.

**Supplementary Information:**

The online version contains supplementary material available at 10.1186/s42466-024-00345-0.

## Background

Blood pressure modification might affect the functional outcome in patients undergoing mechanical thrombectomy due to acute ischemic stroke with relevant vessel occlusions[1‑3]. Much research have focused on the association of preprocedural blood pressure measured during the hospital admission and its U-shaped relationship with worse functional outcome and mortality [4–6], as well the relationship of the postprocedural blood pressure after mechanical thrombectomy with functional outcome [7–15]. However, the optimal intraprocedural management of blood pressure during mechanical thrombectomy is not well established. Guidelines suggestions are based on low to at most moderate level of evidence with recommendations to hold systolic blood pressure (SBP) < 180 mmHg and > 140 mmHg in patients during endovascular stroke treatment (EST), and these boundaries are based on a low quality of evidence [16–20]. 

A general approach to blood pressure management with standardized blood pressure targets might not be optimal for the individual patient suffering from stroke due to their specific collateral status, vessel occlusion sites, cardiac function, penumbral size, or their range of cerebral autoregulation. A higher than needed blood pressure could lead to cerebral edema and hemorrhages and lower blood pressure might enable a faster progression from oligemic to infarcted areas [21, 22]. The compensatory elevated blood pressure during acute ischemic stroke which is measured on hospital admission might be a feasible optimal blood pressure target as this could reflect a level, where the penumbral state is still sufficiently perfused while simultaneously avoiding higher blood pressure than actually required. The aim of this study is to provide further understandings of the relationship between intraprocedural blood pressure during mechanical thrombectomy with clinical outcomes and specifically to determine, whether there is a positive association of time or proportion of intraprocedural SBP spent during the mechanical thrombectomy in a range near the presumably optimal SBP value measured on admission with functional outcome.

## Methods

This is a retrospective study of patients from a single center at a university hospital suffering from acute ischemic stroke undergoing endovascular stroke treatment which were treated between August 2018 to September 2020. Patients treated with endovascular therapy of vessel occlusion of the anterior circulation (internal carotid artery or middle cerebral artery) according to local standard were included into the study. Patients with vessel occlusion of the posterior circulation (vertebrobasilar or posterior cerebral artery strokes) were excluded, as well as patients with recurring ischemic strokes within 3 months or where the anesthetic protocol was not available. The particularity in our center is that peri-interventional anesthetic care was performed by neurologists of the neurocritical care team with standardized protocol-based bundles [23]. For blood pressure management, norepinephrine was used as a vasopressor and urapidil as specific antihypertensive medication. Propofol and Remifentanil were used as main analgo-sedative medication. We applied a systolic blood pressure target of 140 to 160 mmHg for the patients undergoing thrombectomy. Patients were mostly treated with procedural sedation and general anesthesia was only performed when (i) agitation could not be handled with a non-general anesthetic sedation strategy, (ii) there was respiratory insufficiency, (iii) patients vomited or (iv) procedural complications occurred. After the endovascular procedure, patients were mainly transferred to the stroke unit and only transferred to the intensive care unit when they were mechanically ventilated or had cardiorespiratory instability. This study has been approved by the local ethics committee (Ethikkommission der medizinischen Fakultät Heidelberg: S-506/2021). This research received no external funding.

### Data collection

Baseline demographical, clinical, imaging and neuro-anesthetic parameters were collected. Three-month modified Rankin Score (mRS) was collected in a prospective registry at the local site. Blood pressure data were usually obtained via intermittent non-invasive measurements at least every five minutes. Admission or baseline systolic blood pressure was measured on arrival in the emergency department (when emergency department blood pressure was not available we used the ambulance protocol, if neither was available, the first measured value in the angiography suite was used). The intraprocedural period was defined as the timeframe which started with the groin puncture and ended with the last thrombectomy maneuver leading to the final reperfusion outcome. All blood pressure values were retrieved from patient records.

### Exposure variables and primary outcome

Exposure variables were time (in minutes) and proportion of intraprocedural SBP (in percent of intraprocedural time from groin puncture to last thrombectomy attempt leading to final result) spent in a range of ± 10% and ± 20% around the admission SBP. Primary outcome was the occurrence of mRS score 0–2 after 3 months. Categorical mRS after 3 months was also considered as a primary outcome, but the corresponding ordinal logistic regression models used for its analysis did not meet the proportional odds assumption and were hence omitted.

### Secondary analyses

For primary analyses whose exposure showed a p-value less then 0.05, we conducted a secondary analysis by subdividing the target range in values above and below the bSBP.

### Statistical analysis

Baseline characteristics, clinical details and endpoint variables were summarized descriptively using mean, standard deviation, median, interquartile range, minimum and maximum, and absolute and relative frequencies as appropriate.

The primary analyses were conducted by fitting a binary logistic regression model to the dependent variable mRS 0–2 after 3 months with independent variables being the respective exposure variable, baseline NIHSS, dichotomized pre-stroke mRS (0–2 vs. 3–6), mTICI score (0-2a vs. 2b-3), intubation, age, sex. Adjusted odds ratios (aOR), their corresponding 95% profile likelihood confidence intervals as well as descriptive p-values from Z-tests of the regression coefficients were reported.

Intra-procedural SBP data were usually recorded every 5 min. If some points in time had missing SBP values, they were imputed by the mean of the last observed measurement before and the first observed measurement after the missing value.

Missing values in the mRS after three months (20 patients), in the mTICI (5 patients) and in the pre-morbid mRS (one patient) were multiply imputed using the fully conditional specification method with proportional odds and polytomous imputation models. Apart from the variables in the primary analysis model, we also included the covariates mRS at discharge, age and sex in the imputation models. These additional predictors did not have missing values. The regression model estimates on the 50 imputed datasets were pooled using Rubin’s rules.

Spline regression using generalized additive models and B-splines was performed for SBP on admission, mean intraprocedural SBP and maximal deviation from admission level SBP (sum of above and underneath the admission level SBP divided by bSBP) with the probability to reach favorable outcome (mRS 0–2).

### Data Availability

Deidentified participant data can be obtained upon request from Min Chen beginning at 01-01-2025 and ending at 01-01-2030. Every investigator providing a methodologically sound proposal to test the data for confirmation or on a clinically relevant novel research question will be given access to the data repository after positive evaluation of his/her request by Min Chen. Proposals should be directed to min.chen@med.uni-heidelberg.de. After approval, the requestor will have to sign a data access agreement. All documents are available for a predetermined time, typically 12 months.

## Results

**Fig. 1:**
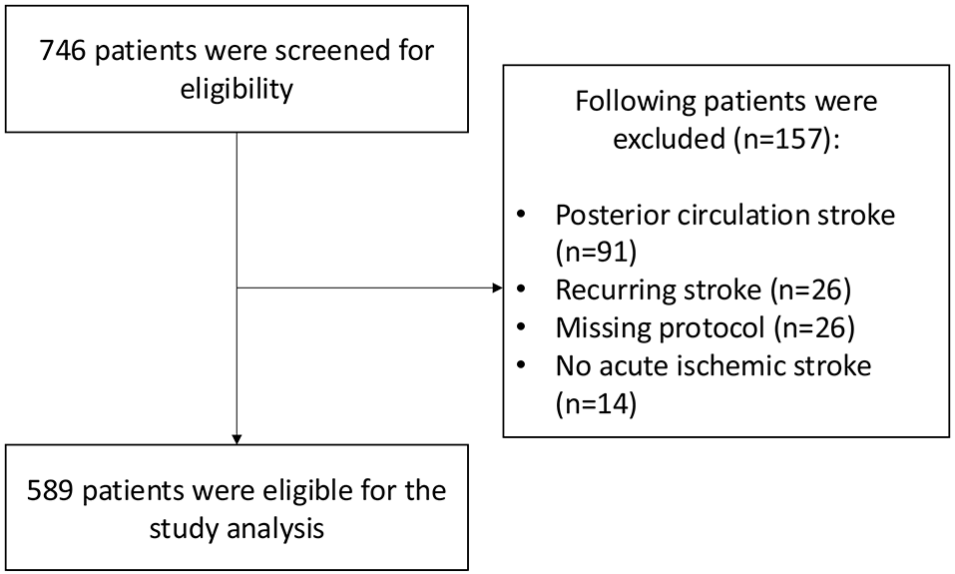
Study flowchart

In this retrospective analysis we screened 746 patients suffering from acute ischemic stroke receiving endovascular stroke treatment from September 2018 to September 2020. From those, 157 were excluded due to different reasons (see Figs. 1) and 589 were included in the analysis.

### Baseline demographics

The mean (SD) age was 76 (12) years and 315 (53%) patients were women. The mean (SD) baseline NIHSS was 15 (7.5) and mean (SD) pre-stroke mRS score was 1.5 (1.4). 248 (42%) patients received intravenous thrombolysis. 469 (80%) patients had pre-existing arterial hypertension. Mean time from onset/last-seen-well to groin puncture was 472 (499) minutes. Other baseline characteristics are depicted in Table 1.

**Table 1:** Baseline clinical and procedural characteristics

Patients characteristics	*n* = 589
Age, mean (SD), y	76 (12)
Sex, n (%)	
Women	315 (53)
Men	274 (47)
Pre-stroke mRS, mean (SD)^a^	1.5 (1.4)
NIHSS, mean (SD)	15 (7.5)
Pre-interventional ASPECTS, mean (SD)^b^	8.2 (1.7)
Medical history, n (%)	
Hypertension	469 (80)
Diabetes mellitus	170 (29)
Hypercholesterolemia	240 (41)
Previous stroke or TIA	134 (23)
Atrial fibrillation	274 (47)
Occlusion site, n (%)	
Internal Carotid Artery	31 (5)
M1 segment of the middle cerebral artery	255 (43)
M2 segment of the middle cerebral artery	138 (23)
Carotid - T	129 (22)
Left side	317 (54)
Treatment, n (%)	
Intravenous thrombolysis	248 (42)
Stenting^c^	105 (18)
Times, mean (SD), min	
Onset / last-seen-well to admission^d^	383 (382)
Onset / last-seen-well to groin puncture^e^	472 (499)
Onset / last seen well to final thrombectomy attempt^f^	537 (515)
Groin puncture to final thrombectomy attempt	72 (56)
Procedural characteristics	
Number of thrombectomy attempts, median (IQR)	2 (2)
General anesthesia, No. (%)	38 (6.4)
Use of vasopressor medication, No. (%)	268 (46)
Use of anti-hypertensive medication, No. (%)	112 (19)

a, missing in 1 patient

b, missing in 174 patients

c, missing in 2 patients

d, missing in 40 patients

e, missing in 38 patients

f, missing in 87 patients

### Blood pressure characteristics

Admission blood pressure was obtained in in 428 cases from the emergency room, in 102 cases from the ambulance protocol, and in 59 cases from the angiography protocol. The mean (SD) baseline SBP was 167 (28) mmHg and mean (SD) intraprocedural SBP was 147 (21) mmHg with a mean difference of -19 (28) mmHg. Mean (SD) minimal and maximal intraprocedural SBP was 128 (25) and 169 (25) mmHg. The mean (SD) time of SBP above the admission blood pressure was 19.3 (33.7) minutes, the mean time below was 53.8 (50.6) minutes. Further blood pressure characteristics are shown in Table 2. The mean (SD) proportion of intraprocedural SBP spent in a range of the bSBP ± 10% was 38 (33) % and in a range ± 20% of the bSBP was 67 (35) %. Number of missing SBP values are depicted in Supplementary Table 1. More parameters are shown in Table 2.

**Table 2:** Blood pressure characteristics

Blood pressure characteristics	Mean (SD)
	*N* = 589
Baseline SBP	167 (28)
Mean intraprocedural SBP^a^	147 (21)
Minimal intraprocedural SBP^a^	128 (25)
Maximal intraprocedural SBP^a^	169 (25)
Baseline MAP^b^	117 (18)
Mean intraprocedural MAP^c^	102 (15)
Minimal intraprocedural MAP^c^	89 (16)
Maximal intraprocedural MAP^c^	116 (18)
Time of SBP in range of bSBP ± 10%, minutes	26 (34)
Proportion of time where SBP was in range of bSBP ± 10%, %	38 (33)
Time of SBP in range of bSBP ± 20%, minutes	46 (46)
Proportion of time where SBP was in range of bSBP ± 20%, %	67 (35)
Difference between mean intraprocedural and baseline SBP, mmHg^a^	-19 (28)
Difference between minimal intraprocedural and baseline SBP, mmHg^a^	-38 (32)
Difference between maximal intraprocedural and baseline SBP, mmHg^a^	2.7 (28)
Difference between mean intraprocedural and baseline MAP, mmHg^d^	-15 (20)
Difference between minimal intraprocedural and baseline MAP, mmHg^d^	-27 (21)
Difference between maximal intraprocedural and baseline MAP, mmHg^d^	-0.14 (21)

a, some values missing in 97 patients

b, missing in 19 patients

c, some values missing in 99 patients

d, some values missing in 115 patients

### Outcome characteristics

Successful reperfusion (defined as mTICI 2b-3) was achieved in 520 (89%) patients after a median (IQR) number of 2 (2) thrombectomies. 29% patients had a mRS score 0–2 at 3 months after the stroke event and mean (SD) mRS score was 3.6 (1.9). 154 (27%) of the patients died during the first 3 months after stroke. General anesthesia was necessary in 38 (6.4%). Other characteristics are shown in Table 3.

**Table 3:** Outcome characteristics

	*n* = 589
mRS 0–2 at 3 months, No. (%)^a^	172 (29)
mRS at 3 months, mean (SD)^a^	3.6 (1.9)
In-hospital mortality, No. (%)	72 (12)
3-month mortality, No. (%)^b^	154 (27)
NIHSS at 24 h, mean (SD)^c^	12 (10)
NIHSS at 72 h, mean (SD)^d^	10 (10)
mTICI 2b-3, No. (%)^e^	520 (89)

a, missing in 20 patients

b, missing in 13 patients

c, missing in 32 patients

d, missing in 224 patients

e, missing in 4 patients

### Primary outcome analysis, sensitivity and secondary analyses

There are higher odds to reach an mRS score 0–2 with higher proportion of intraprocedural time where SBP was around the range of baseline systolic blood pressure (bSBP) ± 20% (aOR 1.007; 95%-CI, 1.0003 to 1.013; *P* = .039), while other exposures did not show any association with favorable outcome (see Table 4).

**Table 4:** Multivariable regression analysis results. An adjusted odds ratio greater than 1 means that the odds for an mRS score 0–2 are higher with increasing time or proportion of time in the specified SBP range

	Adjusted OR [95-% CI]	*P*-value
Time of SBP in bSBP ± 10%, minutes	0.996 [0.99; 1.003]	0.308
Time of SBP in bSBP ± 20%, minutes	0.996 [0.99; 1.001]	0.101
Proportion of time where SBP was in range of bSBP ± 10%, %	1.005 [0.999; 1.011]	0.127
Proportion of time where SBP was in range of bSBP ± 20%, %	1.007 [1.0003; 1.013]	0.039

A sensitivity analysis with ASPECTS as additional predictor variable yielded comparable results (Supplementary Table 2). A secondary analysis of proportion of intraprocedural time where SBP was in the range of 80–100% bSBP or 100–120% bSBP did not show any association with favorable outcome (Supplementary Table 3). Furthermore, sensitivity analysis with thrombolysis as covariable showed, that interaction with the exposure was close to 1 in all models with the smallest p-value being 0.157 for the interaction with time of SBP in bSBP ± 10% [min]. Thrombolytic therapy as an independent covariate did however hint at higher odds for a favorable outcome in the model on time of SBP in bSBP ± 10% [min] (aOR for mRS 0–2 in presence of thrombolysis: 1.866, *p* = .029).

### Spline logistic regression analysis

**Fig. 2:**
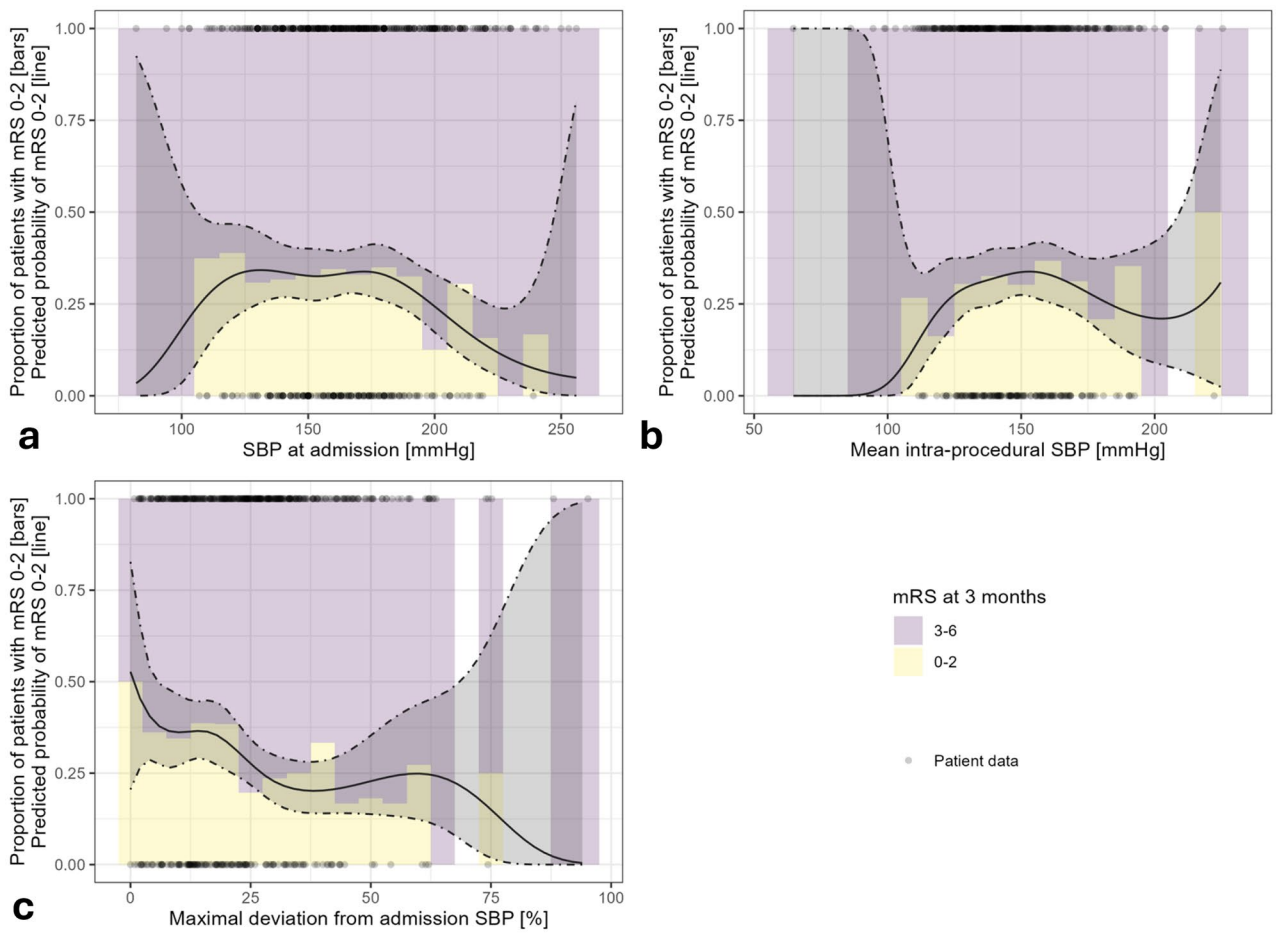
Spline graphs of different blood pressure parameters with probability of favorable outcome (mRS 0–2). **A**, SBP at admission and mRS 0–2 hints at an (inverted) U-shape, however the edges are sparsely represented in the data (*P* = .099). **B**, Spline regression of mean intra-procedural SBP show no significant relationship (*P* = .471). **C**, Maximal deviation from admission SBP show a mostly monotonous decrease of favorable functional outcome with increasing deviation (*P* = .038). Dot-dash lines depict ± 2 standard errors

Logistic spline regression of favorable functional outcome modeled on admission SBP hints at an inverse U-shaped relationship, however the edges are only sparsely represented. The p-value is 0.09928 (see Fig. 2a).

Spline regression of favorable functional outcome modeled on mean intra-procedural SBP mostly shows a constant plateau, perhaps with a small (statistically insignificant) decrease at low SBP values. The p-value is 0.4716 (see Fig. 2b).

Spline regression of favorable functional outcome modeled on relative intra-procedural deviation from admission SBP shows a mostly monotonous decrease of favorable functional outcome with increasing deviation. The p-value is 0.03756 (see Fig. 2c).

## Discussion

In this retrospective study, we investigated whether the admission bSBP poses a useful target value for the intraprocedural blood pressure and whether a larger proportion of intraprocedural time spent in a blood pressure corridor at the level of the bSBP is associated with better clinical outcome.

For that, 589 patients receiving endovascular stroke treatment of vessel occlusions of the anterior circulation were investigated. We were able to find an association of the proportion of time where the intraprocedural SBP was near the admission level SBP (within 80–120%) with better functional outcome.

While there are guideline recommendations with low level of evidence to maintain systolic blood pressure between 140 mmHg and 180 mmHg, such general approach might be suboptimal from a pathophysiological standpoint.[16‑19] There is retrospective evidence that negative blood pressure deviations from the blood pressure measured on admission are associated with worse functional outcome and/or higher mortality. A ΔMAP of 10% between admission and procedural blood pressure was associated with poor outcome [24]. Blood pressure drops from admission levels were found to be detrimental in several other studies [25‑28].

Furthermore, not only blood pressure decreases, but also high blood pressure seems to be associated with worse outcome. One retrospective analysis showed an association of lower intraprocedural SBP with good outcome [29]. Another retrospective study found an association of worse functional outcome with higher intraprocedural mean SBP and MAP [30]. 

There is evidence of a U-shaped relationship between admission blood pressure and functional outcome and worse outcomes are found in patients with low or high admission blood pressure before EST [4, 5, 31]. Similar findings can also be observed for the blood pressure during the EST: A post-hoc meta-analysis of individual patient level blood pressure data from three RCTs investigating the optimal sedation mode for thrombectomy revealed a detrimental association of intraprocedural mean arterial blood pressure which is either too high or too low. Worse outcome was found when MAP was less than 70 mmHg for more than 10 min or when MAP was higher than 90 mmHg for more than 45 min [2]. In a spline regression analysis, we could find a trend toward a (inversed) U-shape relationship between admission SBP and probability of favorable outcome. However, we could not find a convincing relationship between intraprocedural SBP and favorable outcome (*P* = .47), which could be due to the very sparse occurrence of blood pressure at the edges during the thrombectomy procedure.

This is the reason why we assumed, that there might be an optimal blood pressure corridor for stroke patients and the admission blood pressure might be a feasible target to determine the best blood pressure range. Indeed, in the current study, we could find an association of proportion of intraprocedural SBP spent in the range of 20% around admission blood pressure with favorable functional outcome. For every 10% points of procedure time with systolic blood pressure spent in the ± 20% range around the admission blood pressure, the odds for a favorable mRS score of 0–2 increase by 7%. Additionally, the spline graph of maximal deviation of intraprocedural SBP from admission SBP show a monotonous decrease of probability to reach favorable outcome with higher deviations, which further supports the hypothesis, that deviations from admission level blood pressure might have a detrimental association with outcome.

We did not find a significant association in the ± 10% range around the admission blood pressure, however we found a trend for a positive association with favorable outcome and lack of significance could be due to lack of power.

When examining the effects of sustained blood pressure deviations on functional outcome, deviations measured in absolute time in minutes as exposure variable must be interpreted with caution. There is evidence of an association of increasing procedural time with worse functional outcome, which indicates that procedure time is a relevant negative predictor and a strong moderator variable [32]. This might be the reason, why in the current study the aOR of absolute time spent in the specified ranges showed trends towards values < 1, which indicated lower odds of favorable outcome. We have decided to use the construct of proportion of procedure time as exposure variable in our analysis, as this could minimize the confounding character of absolute time.

One prospective, randomized controlled trial, which compared admission bSBP as individual blood pressure targets with standardized SBP target of 140–180 mmHg yielded a negative result [33]. As the actual measured blood pressure profiles were very similar between the two treatment arms in that study, inference on the actual therapeutic potential of differential blood pressure management strategies to influence clinical outcome is not possible. The current study aimed at providing further insights for the suggested hypothesis of individualized blood pressure targets based on the baseline or admission level and provide a positive association of time proportion spent in the admission level range with favorable outcome. However, as most stroke patients present with similar elevated blood pressure, an BP management strategy which aims at pursuing the admission level-based BP target could be obsolete, when comparing to a more general approach with a target systolic blood pressure range of 140–180 mmHg, as most admission BP would be in that range. A more differential approach between an individualized and general approach might have a role in patients presenting with blood pressure differing from the general stroke population (e.g. due to unfavorable collateral status, vessel anatomy, intracranial stenoses) and the role of an individualized approach must be further untangled in these special cases.

Furthermore, two randomized controlled studies currently investigate the individualized use of the admission blood pressure as intraprocedural target value. IDEAL BP was a single center feasibility study, which used MAP as the parameter of interest where the standard group of MAP target of 70 to 90 mmHg was compared to the individualized group with MAP targeted ± 10% of a baseline value. Sixty patients were included, and no significant benefit was seen for the individualized approach [34]. The feasibility criteria were not met in the study, because blood pressure targets were very difficult to achieve and in 54 to 61% of the procedural time outside of the desired range.

DETERMINE is a multi-center trial conducted in France, where MAP was also used as the parameter of interest with an individualized target of ± 10% of a baseline value as study intervention and SBP of 140–180 mmHg as control. The patient recruitment is finished, and publication soon is awaited (NCT04352296) [35]. 

This retrospective study is limited by the inherent biases due to its retrospective character. Generalizability is very limited due to its single center database. As blood pressure variations and their putative effect on clinical outcome modification is estimated to be very small, sample size is always relevant to consider and lack of significant results could be partly due to low test power. As this is an exploratory analysis, no correction for multiple testing was conducted. As we used groin puncture as procedure initiation, BP which may have been iatrogenically brought out of range before the groin puncture from the reference BP was not considered in this analysis, yet might have an impact on outcome.

Strengths of this trial are (i) a large amount of blood pressure data points (in total 8341 recorded blood pressure values), (ii) the streamlined peri-procedural management, which was handled by a highly trained Neurocritical care team and (iii) reduced variability in handling the patients due to its single center design. The use of logistic regression models in combination with multiple imputation enables a relatively unbiased analysis making full use of all available data, thus representing the clinical practice at the study center.

## Conclusion

A higher proportion of intraprocedural time with systolic blood pressure near the admission level is associated with a slightly higher odds of favorable functional outcome. Whether this poses a feasible blood pressure target remains to be further investigated.

## Electronic supplementary material

Below is the link to the electronic supplementary material.

Supplementary Material 1

Supplementary Material 2

## Data Availability

Deidentified participant data can be obtained upon request from Min Chen beginning at 01-01-2025 and ending at 01-01-2030. Every investigator providing a methodologically sound proposal to test the data for confirmation or on a clinically relevant novel research question will be given access to the data repository after positive evaluation of his/her request by Min Chen. Proposals should be directed to min.chen@med.uni-heidelberg.de. After approval, the requestor will have to sign a data access agreement. All documents are available for a predetermined time, typically 12 months.

## Abbreviations

aOR: adjusted Odds Ratio
ASPECTS: Alberta stroke programme early CT score
EST: endovascular stroke treatment
(b)SBP: (baseline) systolic blood pressure
MAP: mean arterial blood pressure
mRS: modified Rankin Scale
mTICI: modified treatment in cerebral infarction
NIHSS: National Institutes of Health Stroke Scale
RCT: randomized controlled trial
