# Supplementary material for: Association of intraprocedural near admission-level blood pressure with functional outcome in stroke patients treated with mechanical thrombectomy
Source: Neurol Res Pract. 2024 Oct 1;6:46. doi: 10.1186/s42466-024-00345-0 (PMC11443703; doi:10.1186/s42466-024-00345-0)
Supplement: Supplementary file 2 — Supplementary Material 2 [file 42466_2024_345_MOESM2_ESM.docx]

**Supplemental Material**

Table 1. Number of missing SBP values

| Number of missing values | No. (%) |
| --- | --- |
| 0 | 492 (84) |
| 1 | 62 (11) |
| 2 | 18 (3) |
| 3 | 6 (1) |
| 4 | 6 (1) |
| 5 | 2 (0) |
| 6 | 2 (0) |
| 7 | 1 (0) |
| Mean (SD) | 0.29 (0.83) |
| Median (IQR) | 0 |

Table 2. Association of time and proportion of intraprocedural SBP with favorable outcome (mRS 0-2) spend in the specified ranges around the baseline SBP with ASPECTS as additional predictor variable as a sensitivity analysis.

Abbreviations: bSBP, baseline systolic blood pressure; SBP, systolic blood pressure

|  | Adjusted OR [95-% CI] | P-value |
| --- | --- | --- |
| Time of SBP in bSBP ± 10 % range, minutes | 0.996 [0.99; 1.004] | 0.325 |
| Time of SBP in bSBP ± 20 % range, minutes | 0.996 [0.99; 1.001] | 0.142 |
| Proportion of SBP in bSBP ± 10 % range, % | 1.005 [0.998; 1.012] | 0.146 |
| Proportion of SBP in bSBP ± 20 % range, % | 1.007 [1.001; 1.014] | 0.03 |

Table 3. Secondary analysis of proportion of SBP either 100 -120 % of bSBP or 80-100 % of bSBP with favorable outcome (mRS 0-2).

|  | Adjusted OR [95-% CI] | P-value |
| --- | --- | --- |
| Proportion of SBP in bSBP + 20 % range, % | 1.005 [0.998; 1.012] | 0.162 |
| Proportion of SBP in bSBP - 20 % range, % | 1.003 [0.996; 1.009] | 0.391 |
